# Plant species classification using flower images—A comparative study of local feature representations

**DOI:** 10.1371/journal.pone.0170629

**Published:** 2017-02-24

**Authors:** Marco Seeland, Michael Rzanny, Nedal Alaqraa, Jana Wäldchen, Patrick Mäder

**Affiliations:** 1 Institute for Computer and Systems Engineering, Technische Universität Ilmenau, Ilmenau, Germany; 2 Department Biogeochemical Integration, Max Planck Institute for Biogeochemistry, Jena, Germany; Nanjing Normal University, CHINA

## Abstract

Steady improvements of image description methods induced a growing interest in image-based plant species classification, a task vital to the study of biodiversity and ecological sensitivity. Various techniques have been proposed for general object classification over the past years and several of them have already been studied for plant species classification. However, results of these studies are selective in the evaluated steps of a classification pipeline, in the utilized datasets for evaluation, and in the compared baseline methods. No study is available that evaluates the main competing methods for building an image representation on the same datasets allowing for generalized findings regarding flower-based plant species classification. The aim of this paper is to comparatively evaluate methods, method combinations, and their parameters towards classification accuracy. The investigated methods span from detection, extraction, fusion, pooling, to encoding of local features for quantifying shape and color information of flower images. We selected the flower image datasets Oxford Flower 17 and Oxford Flower 102 as well as our own Jena Flower 30 dataset for our experiments. Findings show large differences among the various studied techniques and that their wisely chosen orchestration allows for high accuracies in species classification. We further found that true local feature detectors in combination with advanced encoding methods yield higher classification results at lower computational costs compared to commonly used dense sampling and spatial pooling methods. Color was found to be an indispensable feature for high classification results, especially while preserving spatial correspondence to gray-level features. In result, our study provides a comprehensive overview of competing techniques and the implications of their main parameters for flower-based plant species classification.

## Introduction

Although flowering plants play a key role in terrestrial ecosystems, humans increasingly lack the ability for their classification [1]. In addition, the classical way of plant classification, i.e., following a single access identification tree of dichotomous keys, is a complicated and tedious procedure for non-experts. However, due to tremendous achievements in the fields of computer vision and machine learning, automated image based classification promises an easy and fast way for plant classification. Using leaf images for this task was extensively investigated in previous studies, e.g., [2, 3]. Whereas leaves can be found at almost any time throughout a year, the acquisition of suitable leaf images poses difficulties as foreground segmentation is required for extracting discriminative shape parameters. Furthermore, such shape parameters are in most cases only valid for a certain leaf type, i.e., plain single leaves.

The visually most prominent and perceivable part of a plant is its flower, a subject of intense studies by botanists and often the key for species identification. Flowers exhibit great diversity in color, shape and texture, thus allowing to make use of a broad set of methods developed for object classification tasks.

The general difficulty in flower image based plant classification arises from visually small interclass variances in relation to large intraclass variances. Often only little differences in the appearance of visually similar flowers have to be taken into account for accurate classification. The category of such classification tasks is thus termed fine-grained classification. For such tasks the usage of local image features, i.e., a number of image regions corresponding to objects or parts thereof, allows for considerably higher classification accuracies compared to analyzing the whole image content equally. Early studies on flower classification utilized specifically crafted descriptors relying on foreground segmentation and subsequent description of flowers by contour parameters and color histograms, e.g., [4]. Unfortunately such approaches are highly specific and often only applicable to a certain inflorescence type and distinct viewpoint. By using a set of local features and publishing the first benchmark dataset for flower classification, Nilsback and Zissermann pioneered a new research direction that motivated further work towards general purpose descriptors instead of specifically crafted ones [5]. Local features are also used in other computer vision research areas, such as augmented reality [6], 3D reconstruction [7], visual odometry [8], person tracking [9] or mobile visual search and landmark recognition [10–13]. However, only few studies investigated the characteristics and performance of flower classification by local features and those relied on a rather narrow selection of methods [5, 14–21].

Using local features in a machine learning pipeline requires a well-defined sequence of steps and each of those can be realized by a multitude of different methods, thus spanning a large variety in combinations of these methods. The aim of this work is to comparatively evaluate method combinations towards their classification accuracy in flower image based plant classification on three different datasets. Furthermore, by comparing the obtained results on these datasets we show the beneficial use of well-defined constraints during image acquisition.

Our paper is structured as follows: The image classification pipeline using local features is briefly discussed in section *Fundamentals*, followed by a review of related work on flower image based classification using local features in section *Related Work*. By reviewing and discussing comparative studies on object classification in section *Research Scope*, we define the selection of methods investigated in this work. We introduce the datasets used and discuss experimental parameters in section *Methods*. The results are then presented and discussed in section *Results and Discussion* and concluded thereafter.

## Fundamentals

**Fig 1:**
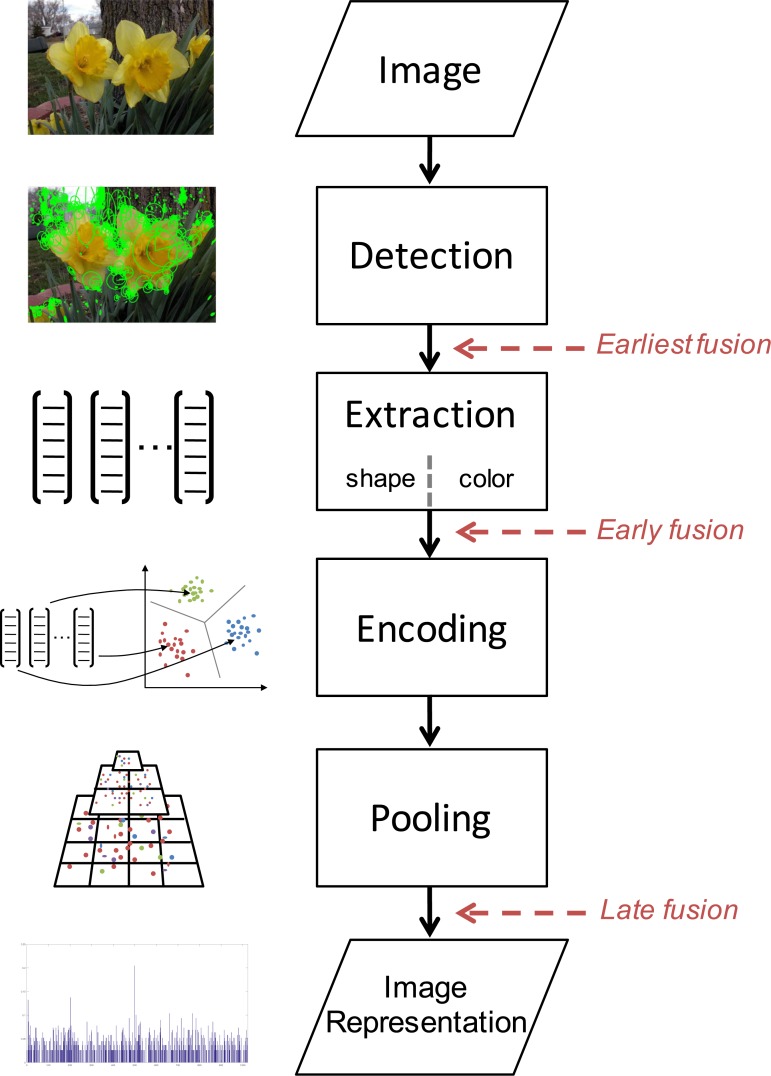
Pipeline for computing image representations based on local features. Local features are computed or defined within the **detection** step. Then local descriptor vectors are **extracted** from these patches and are **encoded** after **pooling** from predefined image regions. The **image representation** is finally used for training and classification.

For training a classifier such as a Support Vector Machine (SVM), it is required to quantify the information contained in every image into vectors of fixed length, i.e., image representations. The dimensions of the image representation span the descriptor space in which the classification process is reduced to a similarity measure between descriptors. However, given a set of images the amount of local features per image can be very different as it depends on the amount of structurally prominent regions as well as the image size. Thus, a processing pipeline is utilized that aggregates the visual information of many local features extracted from an image into an image representation (see Fig 1).

The process typically starts by applying a number of pre-processing steps to an image, e.g., resizing and color space conversion. In the **detection** step, positions of local features are computed following strict mathematical definitions. The image patches surrounding these local features are then quantified using a descriptor extractor within the **extraction** step [22]. In the **encoding** step, local descriptors are aggregated into a global image representation that is used for classifier training and testing along with the corresponding image class label. Using different methods for the previous steps allows for quantification of complementary image information, e.g., separate extraction of shape and color information. **Fusion** of these complementary information can be performed at various stages within the pipeline (see Fig 1). In order to introduce weak geometric context during encoding, local features can be **pooled** from predefined image regions and encoded for forming subimage representations that are concatenated thereafter.

Next, we review the usage of local features for flower based plant classification in previous and related work and will then detail the methods investigated in this work.

## Related work

Whereas in the early days of computer aided plant classification researchers often reported results on unpublished datasets, the publication of the Oxford Flower 17 [5] and Oxford Flower 102 [14] datasets along with splits and accuracy score definitions today allows for comparing results of different studies utilizing different methods. These datasets are an accepted benchmark for fine-grained flower-based classification tasks. We reviewed relevant publications on flower-based plant classification using local features and summarized their methods for each step of the processing pipeline (see Fig 1) sorted by year of publication in Table 1. The table solely lists the methods, but they are briefly described in the next section.

**Table 1:** Results reported for the Oxford Flower 17 and Oxford Flower 102 datasets using local features along with the methods detailed for each processing step.

Study	Det.	Extraction	Encoding	Fusion	Pooling	Seg.	Accuracy [%]		Comments
							OF17	OF102	
Nilsback and Zisserman [6]	pixels	hsv values	BOW (500)	late	1x1	yes	71.8	–	
	dense	SIFT	BOW (1k)						
Nilsback and Zisserman [15]	pixels	hsv values	BOW (500)	none	1x1	yes	–	43.0	only on foreground boundary
	dense	SIFT	BOW (8k)				–	55.1	
	dense	SIFT	BOW (8k)				–	32.0	
	dense	HOG	BOW (1.5k)				–	49.6	
	– above 4 fused –			classifier	1x1	yes	85.1	72.8	multiple-kernel learning
Nilsback [16]	pixels	hsv values	BOW (900)	none	1x1	yes	65.9	44.6	only on foreground boundary
	dense	SIFT	BOW (8k)				72.1	57.5	
	dense	SIFT	BOW (1k)				65.9	34.6	
	dense	HOG	BOW (1.5k)				58.6	50.9	
	– above 4 fused –			late	1x1	yes	87.3	69.4	
	– above 4 fused –			classifier	1x1	yes	88.1	73.7	multiple-kernel learning
Chai et al. [17]	pixels	Lab values	BOW (800)	late	1x1	no	–	64.7	
	dense	SIFT	LLC (8k)						
	dense	SIFT	LLC (8k)			yes	91.1	80.0	
	DoG	SIFT	LLC (8k)						
Liu et al. [18]	dense	SIFT	BOW (1k)	classifier	5x5	no	88.2	–	5x5 overlapping components
	dense	C-SIFT	BOW (1k)						
Angelova et al. [19]	dense	HOG	LLC(8k)	none	1x1+3x3	no	–	76.7	
	dense	HOG FG-HOG	LLC (8k)	late	1x1+3x3	yes	–	80.87	only on foreground
Chai et al. [20]	dense	Lab values	LLC (1k)	late	1x1	yes	–	85.5	
	MSDS	SIFT	FV (256)						
Xie et al. [21]	dense	SIFT	LLC (2k)	none	1x1+2x2+4x4	no	69.5	–	
	dense	Oppo- nentSIFT	LLC (2k)	earliest	1x1+2x2+4x4	no	91.4	–	geometric phrase pooling, spatial weighting
Yang et al. [22]	MSDS	SIFT	BOW (600)	none	1x1+2x2+4x4	no	72.6	42.6	
	MSDS	SIFT	BOW (600)	late	1x1+2x2+4x4	yes	78.3	49.1	SIFT on foreground and whole image, multiple-kernel learning
				classifier			79.3	51.0	

### Detection

A large portion of previous work relied on the whole image content and used it equally for classification, often after classifying the foreground region either manually or automatically by segmentation. Using segmented images improved the overall reported accuracies in all cases, e.g., [16, 18, 21] in Table 1. Studies on unsegmented images often incorporated a weighted pooling strategy, e.g., by using a saliency based spatial weighting scheme for local features [20, 21]. Apart from treating every pixel equally, e.g., for extraction of color information [5, 14, 16, 19], a descriptor extractor quantifies image information of a patch surrounding a local feature. According to Table 1, a dense definition of local features, i.e., intersection points of a grid with constant step size and patch sizes chosen to generate overlapping patches, was used in every publication. The multi-scale dense sampling (MSDS) is an extension of the dense sampling by varying step and patch size. Only Chai et al. incorporated the Difference of Gaussians (DoG) as detector for local features along with pixel-based and dense sampling [16].

### Extraction

For descriptor extraction, i.e., quantifying the content of image patches, the Scale-Invariant Feature Transform (SIFT) [23] and the Histogram of Oriented Gradients (HOG) [24] were previously studied as general purpose shape descriptors extracted from grayscale images. In [14], HOG allowed for an accuracy of 49.6% on segmented images of the OF102 dataset and was thus used as complementary descriptor in combination with SIFT and color values. Interestingly, [18] used HOG as sole descriptor in combination with a huge codebook (8k codewords) and gained exceptionally high accuracies, even on unsegmented images (76.7% on unsegmented images). In contrast to this result, Nilsback et al. ([14] and [15]) studied HOG with optimized codebooks (250 to 4k codewords) and found the classification accuracy of HOG significantly lower than that of SIFT and a decreasing accuracy for codebooks larger than 1.5k codewords.

Two different strategies were used for the extraction of color descriptors: (1) assessing the color values of all foreground pixels in a color space allowing for adequate color quantification, i.e., hsv [5, 14, 25] or Lab color space [16, 19], and (2) using SIFT variants that concatenate SIFT descriptors computed on every channel of an input image. The best performing SIFT variants are either computed in opponent color space or normalized opponent color space, i.e., OpponentSIFT and C-SIFT [26].

### Encoding

The most simple yet often successful approach for computing an image representation out of local feature descriptors is the “bag-of-visual-words” (BOW) approach that was applied by the majority of previous work (see Table 1). As the hard-assignment during BOW encoding inherently causes information to be lost, soft-encoding variants such as the Locality Constrained Linear Coding (LLC) were often used after its initial publication by Wang et al. [27]. In [19] the Fisher Vector was used to encode MSDS-SIFT descriptors sampled on the foreground area of segmented images.

### Fusion

For fusion of color and shape information, very different approaches were used so far. The most simple thus often used strategy is concatenation of the image representations computed for every feature, e.g., pixel-based hsv values and patch-based SIFT descriptors after encoding [5, 16, 19, 21]. In this paper this fusion method is referred to as “late” fusion as the fused image representation is obtained at the latest stage before feeding the classifier. Using SIFT variants like OpponentSIFT and C-SIFT, e.g., [17, 20], color information is stored automatically within the shape descriptors computed on each color channel and concatenated thereafter. As color and shape information are thus combined at the earliest possible stage is referred to as “earliest” fusion in this publication.

Several publications report on fusion at the classifier level, e.g., by training one SVM per feature type and using the prediction score averaged across all SVMs for the overall prediction [17]. Advanced classifier fusion strategies employ methods such as multiple-kernel learning (MKL), where a linear combination of SVM kernels [28], one for each feature, is learned [14, 15, 21]. In Table 1 this strategy is referred to as “classifier” fusion. We do not study this fusion option within our study since we aim to solely investigate the discriminative power of local features while keeping the effect of the classifier constant. Apart from the publications reported in Table 1, more publications on classifier fusion exist. However, these works, e.g., [28–30], rely on the original descriptors as provided by Nilsback and Zissermann in [14]. As we intend to compare methods with respect to local features while keeping the classifier unaltered, these publications are out of scope for our work and not included in Table 1.

### Pooling

All encoding methods inherently suffer from loss of geometrical information, i.e., the spatial arrangement of local features or image parts during encoding. The missing correspondence between low-level local features and the final image representation is referred to as semantic gap. The most prominent method for filling this gap is to partition the image into non-overlapping subimages of constant size and to pool local features from these subimages. For every subimage an encoding step is performed followed by the concatenation of the subimages’ representations. Combining increasingly fine image partitions forms a spatial pyramid as final image representation [31]. Using a pyramidal kernel results in a dimension for the final image representation that is multiplied by the total number of subimages. Hence, a three-level representation is 21 times larger compared to a one-level global encoding and accordingly requires more time for computing. Among the work in Table 1, Angelova et al. [18] represented the image by 2 layers, i.e., a 3x3 grid in addition to the full image (1x1). Yang et al. as well as Xie et al. combined 1x1, 2x2, and 4x4 image partitions to form a 3 layer image representation [20, 21]. Nilsback [15] compared the one-level image representation to a three-level pyramidal image representation, i.e., 1x1, 2x2, and 4x4. Interestingly, the overall classification accuracy was found to decrease with pooling from 73.7% to 70.1% on the OF102 dataset.

### Notable extensions

Some of the reviewed studies (see Table 1) propose very specific methods in addition to the pipeline displayed in Fig 1. These additions mostly refer to more sophisticated pooling solutions and are reviewed here in a nutshell for completeness of the overview. Liu et al. performed classification on image components, i.e., 5x5 sets of equally sized but overlapping image regions, and trained linear combinations of these component-classifiers for predicting image class labels [17]. Xie et al. [20] aggregated local features neighboring in the image domain by Geometrical Phrase Pooling (GPP) in addition to representing the image by a three-level spatial pyramid. The GPP is an intermediate step that forms mid-level structures aiming to connect low-level features and high-level concepts. Furthermore they used a spatial weighting scheme that favors local feature pooling from sharp image regions by computing edge-maps followed by gaussian blurring.

## Research scope

Given the overview on previously studied methods for each processing step (see Fig 1) in section *Related Work*, we conclude that various general purpose features and methods were successfully used for flower classification so far. However, given the developments of the past decade, many more methods were developed and successfully used, e.g., for general object or scene classification tasks. In this section we review and shortly explain prominent methods for the same steps of the processing pipeline.

### Detection

In general, a local feature is a point or region in an image with a well-defined position in image space, found by a strict mathematical definition, and ideally being stable under local and global deformations, i.e., changes in illumination, orientation, and scale [22]. The underlying assumption of such local features is that visually similar objects exhibit similar or comparable features as long as the same method is used for their detection. However, finding correspondences is unreliable in the presence of geometric and photometric deformations as they affect the shape of such regions. Also changes in the scale of observation impact the appearance of objects in images and thus the size of the regions. Therefore, research on local feature detectors motivated the development of a huge pool of detectors and the demand for benchmarking them. Unfortunately, there is no clear theory allowing to choose which features are most relevant for a particular problem. Hence, we review recent developments and discuss benchmarks performed so far, in order to select the most promising candidates.

Local feature detection is a low-level image processing operation, which can be divided into four main categories based on the resulting types of local features: edge, corner, blob, and region detectors. Edges are connected points in an image where pixel intensities exhibit discontinuities and thus large gradient values. Edges by themselves are rather unsuitable as local features since their appearance varies with image scale and rotation. Corners are an intersection of two edges, where points are characterized by the two different edge directions in their local neighborhood. Blobs are image regions either darker or brighter than their surroundings. Regions, the last category, are areas of the image characterized by uniform distribution of pixel intensities or delimited from their surroundings by significant changes in intensity or texture.

Comprehensive reviews of relevant detection methods and their chronological evolution along with computational details can be found in [22, 32–36]. Comparative evaluations of local feature detectors confirm on their repeatability and matching performance, as first introduced by the framework of Mikolajczyk et al. [37].

We found the following results and comparisons relevant for the selection of detector candidates for our study. Comparing detector-descriptor combinations, it was shown that SIFT (Scale-Invariant Feature Transform) is still the best descriptor for arbitrary local feature detectors and object categories or scenes [32, 34, 38–40]. It was observed that SIFT performs bad in combination with its own detector, the DoG (Difference-of-Gaussians) [34]. Instead of DoG, the FAST (Features from Accelerated Segment Test) detector allows for a high number of matches [35] but performs bad under scale variations [41]. Contrary to its high matching performance and robustness under changes in viewpoint, lightning, and scale, MSER (Maximally-Stable Extremal Regions) performs bad for classification problems as typically only a small number of local features are detected [42]. Furthermore, the matching performance of MSER is weak for non-planar scenes or 3D objects [32, 38, 41]. However, MSER as region detector is complementary to corner or blob detectors [41] and it might still be useful in conjunction with other detectors. The HessAff (affine covariant Hessian) detector is often reported to achieve best results due to its robustness against changes in viewpoint, lightning, and scale [32, 34, 37, 38] and slightly outperforms the DoG detector [40]. DoG still offers a better tradeoff between performance and speed [43] as the affine shape estimation of HessAff requires additional geometric and photometric normalization. It was also reported that the matching performance does not or only marginally benefit from affine shape estimation [41]. In [41] it was concluded that a fixed scale Harris corner detector performed better than the Hessian detector. Unfortunately, [42] did not include their own HessAff detector into their comparative evaluation of matching performance using different object classes. Amongst the investigated detectors (DoG, HarrLap, HessLap, and MSER), the HessLap (Hessian-Laplace) was found to perform best. Motivated by these contradicting results, the following research question is proposed:

**Research Question 1 (RQ1)**

Do affine-covariant image patches yield higher classification accuracies compared to their affine-variant counterparts?

In general, detectors providing a large number of local features show better performance for classification problems [34, 40]. This justifies the success of dense sampling and MSDS (multi-scale dense sampling) relying on a grid-based definition of local points instead of their detection. MSDS was found to outperform any local feature detector in scene classification but was severely affected by changes in scale and orientation [34], thus rather unsuitable for classification of 3D objects under different viewpoints, such as flowers. Furthermore, given typical values for image size (500x500 pixels) and step size (5), dense sampling results in 10k local patches to be quantified and encoded. True feature detectors yield about one order of magnitude less patches, thus decreasing the overall time required for computing an image representation. Considering the related work Table 1, a true feature detector was used in only one out of nine studies, whereas dense sampling or MSDS were used in basically all studies although rather unsuitable for flower images of different scales and viewpoints. Hence we propose the following research question:

**Research Question 2 (RQ2)**

Can the vast amount of local features computed by MSDS outperform other local feature detectors?

Motivated by these results, we selected a set of local feature detectors to compare their performance for our classification task by combining them with suitable descriptors. For corners, we chose the recent FAST as well as the Harris detectors, both in a multi-scale variant motivated by their scale dependency. Regarding blobs, we compare the DoG and the Hessian-based detectors including the scale-normalized determinant of the Hessian (DoH) computed from Haar wavelets, as used as detector for the SURF descriptor [44]. MSER is solely used and evaluated as a region detector. In order to evaluate the relevancy and possible benefit of affine covariance, we compare both the Harris and the Hessian with and without affine shape estimation.

### Extraction

Once local features are detected, feature descriptors quantifying the visual information of the patch surrounding the local features are extracted. Extraction methods such as SIFT, SURF, and HOG compute local descriptors based on a gray-level patch, thus quantifying the shape information surrounding a local feature. Detection and descriptor extraction of local features are often treated interdependently, i.e., most detection methods come along with their own description method, e.g., SIFT (DoG) [23] and SURF (DoH) [44]. Similarly to local feature detectors, a large number of descriptor extractors was developed within the past years and their performance was assessed through studies [32, 38–40, 42]. Drawing a general conclusion from these studies, Lowe’s SIFT is still accepted as the most effective gray-level descriptor. This observation is still justified in recent studies, even a decade after SIFT’s development and original implementation [34, 35, 43]. Some studies report that SURF shows performances comparable to SIFT while being several times faster as the computation is performed on integral images [45]. To summarize the SIFT descriptor extraction pipeline in a nutshell, local gradient magnitudes are computed on 4 × 4 sub-regions from the patch around the local feature. The orientation of the gradients of each sub-region is then quantized into an 8 bin histogram, thus forming an 128 dimensional descriptor. The SURF descriptor also relies on 4 × 4 sub-regions but computes the sum of Wavelet responses in horizontal and vertical direction, overall forming a 64 dimensional descriptor. The HOG was first introduced by Dalal and Triggs for pedestrian detection in combination with a Support Vector Machine as a sliding window detector [24]. It works as shape descriptor by binning local gradient magnitudes into an histogram of edge orientations. Typically, HOG is computed on a dense grid of uniformly spaced cells on an image, but it can also be utilized for quantifying the patch appearance around interest points. Given these different methods, the following research question is defined:

**Research Question 3 (RQ3)**

Which shape descriptor (SIFT, SURF, and HOG) allows for the highest flower classification accuracies?

Instead of such real-valued descriptors, binary descriptors such as ORB [46] are considered an efficient alternative as their computation is faster and requires less memory. They achieved high performance in local feature matching applications where pairs of corresponding local features are searched [45, 47]. Only few studies report on their performance in object classification tasks using an encoding pipeline similar to our study [34, 48–51]. These studies draw the following conclusions: binary descriptors are highly suitable for real-time applications or those being executed on low-end and embedded hardware, but their classification performance is still lower than those of real-valued descriptors like SIFT. Based on this general finding, binary descriptors were not evaluated within this study but listed here for completeness.

Apart from shape, the color is the most visually perceivable feature of a flower [52]. In order to utilize this information for flower classification, we consider different methods for the extraction of color features are reviewed. A comprehensive overview on color descriptors is given in [26]. The requirements for a good color descriptor are to be discriminative while possessing photometric and geometric robustness to some degree as well as to be compact [53]. Similar to shape descriptors, several color descriptors are proposed in literature and the best performing ones were considered for our comparative study.

Finlayson et al. presented a Comprehensive Color Image Normalization (CCIN) exhibiting invariance against photometrical distortions and being usable as global image representation [54]. The photometric invariance of CCIN is obtained by iterative normalization of each rgb color channel by their spatial averages after normalization of each rgb pixel based on its channel intensities. Van de Weijer and Schmid adopted the idea of CCIN for local color feature extraction from the patches around interest points [55]. The authors proposed local histograms of photometric invariants as color descriptors being robust to changes in shadowing, shading, specularities and changes of the light source. Results on classification tasks indicated that Robust Hue Histograms (RHH) and Opponent Angle Histograms (OppA) perform equally or better to CCIN. They also found that their color descriptors achieve different results depending on the color saturation within the specific dataset, i.e., the RHH performs better for datasets with saturated colors whereas the OppA is advised for datasets with less saturated colors. The RHH is a hue-histogram where each sample is weighted by its saturation in order to increase robustness of the hue certainty [55]. Similarly, the OppA is weighted with its gradient strength for improving certainty. Whereas previous research in color descriptor design was motivated by imposing photometric invariance, Van de Weijer et al. [56] adopted the Bag-of-Words encoding (detailed in section *Encoding*) for pixel-based conversion of Lab color values of the patch surrounding a local feature to a basic set of 11 color names trained on Google Image queries. The frequency of these color names is then used as descriptor. As different shades of a color are mapped to the same color name, the learned partitioning of the colorspace into 11 clusters automatically induced photometric invariance to a certain degree. Khan et al. additionally improved this color description by clustering bins in Lab space based on their discriminative power in classification tasks on multiple datasets. They learned a universal color descriptor, named Discriminant Color Descriptor (DCD), that can be applied to previously unseen data without the need of learning dataset specific color codebooks [53]. Based on the identified and briefly introduced extraction approaches, our next research question is defined as:

**Research Question 4 (RQ4)**

Which photometric invariant color descriptor is most stable against illumination changes while allowing for high flower classification accuracies?

### Encoding

For classification tasks, the extracted local feature descriptors are encoded into an image representation. The encoding step is based on a codebook often termed “visual vocabulary” following the idea of document classification in text processing [57]. In order to compute this codebook, local feature descriptors are extracted from each training image and are then clustered into *K* clusters using K-means or a Gaussian-Mixture-Model. The totality of cluster centers (aka codewords) defines the codebook, i.e., the vocabulary of “visual words”.

The most simple yet successful approach for computing an image representation is to assign every local feature descriptor to the cluster center nearest in descriptor space, followed by computing the frequency of occurrence of the codewords inside the image. This approach results in a sparse vector of length *K* of the frequency of the codewords (local image features) repeated in a document (image) and is thus often referred to as “bag-of-visual-words” (BOW) [58]. Due to this hard assignment to a single codeword, the BOW suffers from codeword uncertainty, i.e., in cases where distances between a feature descriptor and an adjacent codewords are somewhat comparable, as well as codeword plausibility if the overall distance becomes too large, making the assignment implausible at all. For addressing these problems, soft assignment variants of the BOW that store additional statistics apart from codeword frequencies were developed. The earliest of these methods is the Kernel Codebook encoding (KCB) that models the similarity of local feature descriptors and codewords using a Gaussian kernel [59]. Depending on the kernel parameters every descriptor then contributes some information to every bin of the word occurrence histogram of size *K*. The improvements of such distance-based soft assignment suggested the locality of the codewords being used for encoding local feature descriptors. This is accounted for by the Locality Constrained Linear Coding (LLC) that projects every feature descriptor into a local coordinate system spanned by *M* < *K* codewords closest to the feature descriptor, respectively [27]. Every local feature descriptor is encoded into a sparse K-dimensional vector that contains only zeros except for the *M* components corresponding to the closest codewords. For generating the global image representation of size *K*, the projected coordinates of all local feature descriptors are combined by max-pooling.

Rather than only aggregating codeword occurrences, advanced encoding methods such as the Vector of Locally Aggregated Descriptors (VLAD) [60] and the Fisher Vector (FV) [61] encoding store additional statistics between codewords and local feature descriptors. Applying VLAD, local feature descriptors are encoded by averaging their residuals with respect to the *K* cluster centers. The VLAD encoded image representation is then obtained by concatenating all *K* residuals. The L2 intra-normalization of the image representation, computed by dividing each residual by its norm, was shown to further improve this encoding method [62]. As only the codeword centers are required for the VLAD encoding, the same codebooks as for the BOW and its soft-assignment variants can be used. Given *D* as length of the local feature descriptor, VLAD encoding results in an image representation of length *D* ⋅ *K*. The FV encoding relies on storing the mean and covariance deviation vectors for each component *k* of the Gaussian-Mixture-Model (GMM) and each element of the local feature descriptors [61]. The image representation is computed by concatenating all mean and covariance vectors, resulting in a vector of length *D* ⋅ *K* ⋅ 2.

Chatfield et al. [63] performed a comparative evaluation of the encoding methods mentioned above (VLAD excluded). They used MSDS-SIFT and linear SVMs on top of the different encoding methods utilizing distinct vocabulary sizes. The comparison was performed on the PASCAL VOC 2007 and Caltech-101 datasets. They found that all encoding methods considerably gained from large (25k codewords) codebooks, resulting in correspondingly large image representations. Furthermore the FV outperformed the other encoding methods. However, their comparative evaluation was based on distinct codebook sizes instead of distinct global image representation lengths. 8k and 25k element encodings were compared to 41k element encodings using the FV, whose length is factorized by the local feature descriptor lengths times 2.

The introduced and briefly discussed methods motivated the following research question:

**Research Question 5 (RQ5)**

Do first and second order feature-codebook statistics positively impact the classification accuracy and what are the minimum codebook sizes required for outperforming BOW as baseline?

### Fusion

**Fig 2:**
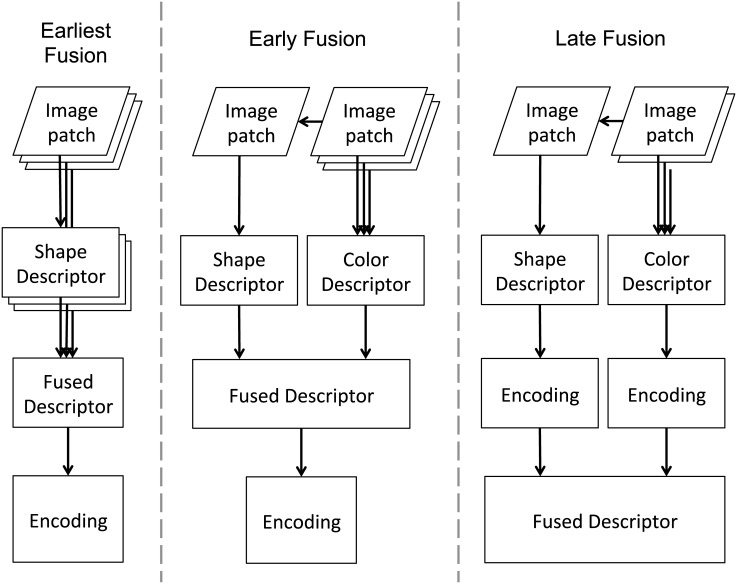
Earliest vs. early vs. late fusion of shape and color features. In the earliest fusion strategy all local shape descriptors are computed from every color channel, followed by concatenation and encoding. In the early fusion strategy, all local features (shape and color) are extracted from the same patches and are locally concatenated before encoding whereas in the late fusion the image representations are computed separately for each feature and concatenated thereafter.

The local shape features discussed in section *Extraction* are traditionally computed on grayscale images. The exclusion of color information is motivated by large variations in color due to perturbations in the illumination conditions, making the task of robust description of color features difficult. However, color is a major feature of flowers and highly beneficial for classification purposes. We thus evaluate three possible strategies for fusing local shape and color features. Previous work [43] categorizes two general strategies: Early Fusion and Late Fusion (see Fig 2).

In early fusion, color and shape are locally combined and then processed together throughout the rest of the classification pipeline [64]. Typically one local shape descriptor is computed for each channel of the color image and all local descriptors are then concatenated. We term this approach ‘earliest fusion’ in order to distinguish it from another early fusion approach. Previous studies found OpponentSIFT descriptors (earliest fused SIFT descriptors in opponent colorspace) to offer superior performance for object classification tasks, such as the PASCAL VOC 2007 [26]. The length of the earliest fused local feature descriptor is three times the length of the respective grayscale descriptor. This is crucial if VLAD or FV is applied for encoding, whereas for the BOW encoding the length of the image representation remains constant, only the memory consumption during codebook generation is multiplied as the local descriptor length increases. For BOW the number of codewords should be increased as the codewords become more discriminative by incorporation of color.

Van de Weijer and Schmid proposed an approach that is termed ‘early fusion’ for the rest of this paper (see Fig 2). It relies on extracting color feature descriptors from the patches used for computing shape descriptors and concatenating both feature descriptors locally [55]. Instead of being multiplied by the number of color channels, the length of the early fused local feature descriptor is the sum of the length of the respective local feature descriptors before concatenation. By fusing the local feature descriptors and mutual encoding, spatial correspondence can be preserved, thus increasing the discriminative power of the local features.

For the late fusion strategy, one image representation is computed per feature type, i.e., local shape and local color features are computed and encoded separately and concatenated thereafter. As spatial correlation is lost by late fusion, the resulting image representations are expected to be less discriminative. Bianco et al. compared earliest and late fusion for pairwise feature matching on several datasets [43]. Their findings implied that the performance of different fusion techniques depends on the dataset. This motivated the following research question:

**Research Question 6 (RQ6)**

How important is the spatial correspondence of features extracted from flower images when performing feature fusion before classification?

### Pooling

Encoding inherently causes information on the spatial arrangement of local features to be lost. The most prominent method of introducing weak geometric information is to partition the image into non-overlapping subimages of constant size per level. In addition to the 1x1 image on the first level, i.e., the original image, the partitioning is repeated on multiple levels, e.g., 2x2 on the second level, 4x4 on the third level [31]. One encoding step is performed for every subimage and all subimage representations are concatenated after local normalization. The dimension of the final image representation is thus multiplied by the total number of subimages, e.g., 21 times for a three level pyramid, hence enlarging the memory footprint accordingly. In the original approach by Lazebnik et al. [31], the subimage representations are normalized according to their size (in the image domain) prior to concatenation. However, according to the original publications of the encoding methods (see section *Encoding*), the L1 norm should be used in case of BOW and KCB whereas the L2 norm is used for LLC, VLAD, and FV. Also Chatfield et al. found this normalization scheme to perform better than region-size dependent normalization [63].

During the encoding of each subimage, local features of the respective region can be pooled in two different ways: (a) using sum-pooling, i.e., the encodings of the local features are combined additively, and (b) using max-pooling, i.e., each bin in the resulting representation is assigned the respective maximum in that region [63]. Typically, max-pooling is used for the LLC encoding and sum-pooling for the other methods [63].

As this spatial binning along with feature pooling became a standard method in image classification, it is widely used for computing improved image representations with weak geometric context (cp. Table 1). In [15] Nilsback compared the standard one-level representation, i.e., 1x1 sum-pooled BOW encoding, to a three-level pyramidal representation, using sum-pooling along with region-size dependent normalization as in [31], and found the classification accuracy to be reduced on the OF102 dataset. Using FV encoding with a large SIFT codebook (256 elements), also Chai et al. achieved remarkable classification accuracy without any spatial binning on the OF102 dataset (see Table 1). Based on these results, we experimentally evaluated to which extent the pyramidal image representation along with respective feature pooling benefits the classification accuracies on the different flower datasets used in this study for answering our last RQ:

**Research Question 7 (RQ7)**

What benefits classification accuracy more: additional feature-codebook statistics or weak geometric context?

## Methods

In the previous section, seven research questions (RQ 1–7) were defined. These questions are answered by evaluating the introduced methods on three datasets: the Oxford Flower 17 (OF17), [65], the Oxford Flower 102 (OF102) [66], and our own Jena Flowers 30 (JF30) [67]. Next, we describe these datasets.

### Datasets

**Fig 3:**
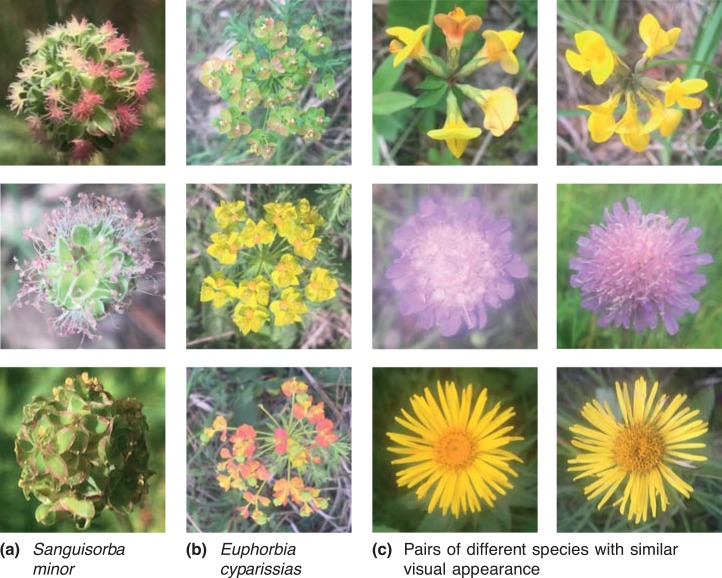
Challenging examples from the Jena Flower 30 (JF30) dataset. (a) and (b) Evolution of two flowers throughout the season and (c) Species with similar visual appearance: *Lotus corniculatus* vs. *Hippocrepsis comosa*, *Scabiosa columbaria* vs. *Knautia arvensis*, *Inula hirta* vs. *Inula salicina*.

OF17 is known as a challenging dataset with 17 flower classes that were chosen to be indistinguishable solely by color. The images have been acquired by searching the web and selecting images of a species with substantial variation in shape, scale, and viewpoint [5]. Each class is represented by 80 images in the dataset. The OF102 is a dataset containing 102 classes represented by 40 to 258 images per class and 8,189 images in total. About 45% of the OF17 images are also part of OF102, i.e., OF17 is not simply a subset of OF102. OF102 is particularly challenging due to both small inter-class variances and large intra-class variances [14]. Our experiments were also performed on our own Jena Flower 30 dataset with 30 classes based on common wild-flowering species found on semi-arid grasslands around the city of Jena in Germany [68]. Classes are represented by 11 to 70 images with a total of 1,479 images. All images were acquired as top-view flower images using an iPhone 6 throughout an entire flowering season. Different illumination conditions were enforced by image acquisition under shaded conditions with enabled and disabled flash as well as through direct exposition to the sun. The dataset is challenging since multiple species exhibit large visual similarities and due to the fact that it covers a variety of blooming stages, drastically impacting the appearance of the flowers. Sample images are shown in Fig 3. The JF30 was added to the evaluation due to its image properties orthogonal to the Oxford Flower datasets: viewpoint and scale are fixed, the flower is in the center of the image and covers about 50% of the image area whereas illumination conditions and intra-class flower appearances are very different. Using these three datasets allows us to investigate the performance of the methods for different sets of conditions.

### Training, testing and implementation details

All classification experiments were performed using a linear Support Vector Machine (SVM), one of the most popular and generalized types of discriminate classifiers [69, 70]. In our experiments the VLfeat implementation [71] was used. Training was performed in a 1-vs-all manner and a *χ*^2^ kernel was applied for linearization of the descriptor space. The regularization parameter was optimized on the validation set by grid search, respectively. In order to quantify classification accuracy, the top-1 prediction per image was evaluated and averaged across all classes.

**Fig 4:**
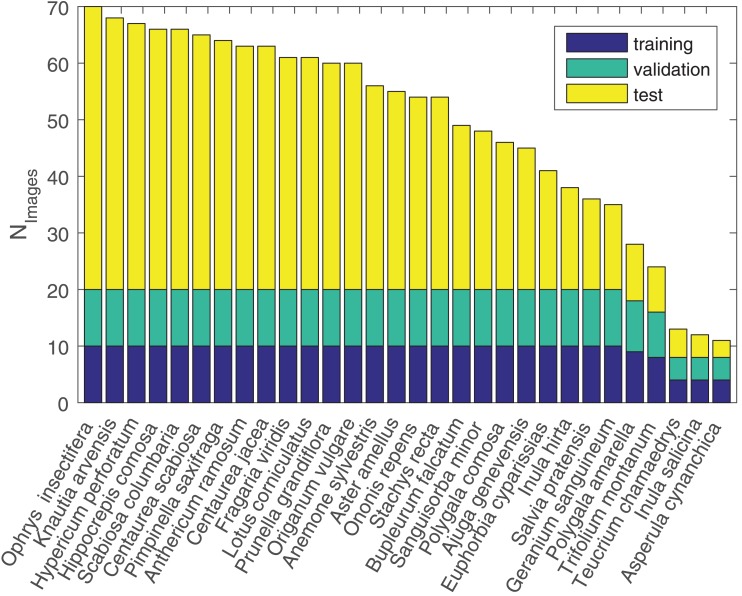
Images per species within the Jena Flower 30 (JF30) dataset.

Training, validation, and test splits were utilized for all three datasets. For the OF17 dataset, every experiment was cross-validated using the three data splits originally provided by the creators containing 30 training, 30 validation, and 20 test images per class. The OF102 dataset is solely divided into training and validation (10 images per class, respectively) and test sets (at least 20 images per class) in a single split, hence no cross-validation could be applied. For the JF30 dataset, three random training (up to ten images), validation (up to ten images), and test splits (remaining images) were created. Whereas 25 classes of JF30 are represented by 35 to 70 images (see Fig 4), five classes are underrepresented by only 11 to 28 images, adding additional difficulty to the dataset.

All experiments were performed on unsegmented images. Although segmentation often improves classification results (compare with Table 1), no use of any object regions or segments was made for classification purposes, even if provided with the datasets. The incorporation of image segmentation adds an additional pre-processing step that is again prone to optimization. While a positive impact on classification rates is expected, we hypothesize that this is not interdependent with the selection and configuration of image descriptors and should therefore impact them equally. Furthermore, in a natural environment, plants of a certain class often live in the same habitat type with comparable visual appearance. Despite clutter, the background might therefore contain useful information for the purpose of plant classification.

For detecting DoG, DoH, FAST, MSER, MSDS features as well as extracting SIFT, SURF, and HOG descriptors the OpenCV implementation, version 2.4.13 [72], was used. HOG descriptors were extracted using three different patch sizes, i.e., 16x16, 24x24, and 32x32. Angelova et al. computed HOG descriptors for four different patch sizes [18]. However, decreased classification accuracies were found in our experiments. Detection using Hessian and Harris variants was performed using the VLfeat library, version 0.9.20 [71]. The RHH, OppA, and DCD color descriptors were computed using the original implementation by van de Weijer [53, 55]. SIFT and DCD descriptors were L1-normalized and square-rooted before encoding [73]. All images were resized to fit a maximum size of 1000 px at either side, except for HOG were a maximum size of 500 px was used as no improvement for larger image sizes was found. BOW encodings were L2 normalized before applying the *χ*^2^ kernel. VLAD and FV encoding was performed using VLfeat and the improved FV including Hellinger’s non-linear additive kernel [74] as well as L2 intra-normalization for VLAD [62] were used. For the encoding experiment, the implementation provided by Chatfield et al. was used for KCB and LLC [63]. For KCB, *K* and *σ* were set to 5 and 100, respectively, whereas for LLC the regularization parameter was set to *β* = 5*e* − 3 and *K* = 11.

### Experiments

Based on the methods identified and briefly introduced in section *Research Scope*, we conducted a set of experiments for answering our research questions. Starting with feature detection methods, we investigated the median number of detected features per image using DoG, DoH, FAST, MSER, MSDS as well as Hessian and Harris along with their Laplacian variants. Next, we compared classification accuracy achieved using Hessian, HessLap, Harris, and HarrLap with and without affine shape estimation. Evaluating these results, we reduced the list of feature detectors and evaluated their classification accuracies in combination with shape descriptors, i.e., SIFT, SURF, and HOG, as well as color descriptors, i.e., RHH, OppA, and DCD. We then evaluated encoding methods, i.e., BOW, LLC, KCB, VLAD, and FV, in terms of classification accuracy and memory footprint using discrete codebook sizes and image representation lengths. Based on the identified best performing detector-descriptor-encoding combination, we compared fusion strategies by using OpponentSIFT (earliest fusion), local concatenation of SIFT and DCD before mutual encoding (early fusion), and global concatenation of SIFT and DCD after separate encoding (late fusion). At last, we evaluated to which extent a spatial pyramid image representation benefits classification accuracy comparing two (1x1, 2x2) and three (1x1, 2x2, 4x4) layer pyramids to the one layer baseline. By comparing the dimensions of the final image representations we investigated the tradeoff between novel encodings (e.g., FV), codebook sizes, and pyramidal image representation.

## Results and discussion

### Detection

#### Amount of detected features

We evaluated the selected local feature detectors by analyzing the median amount of features detected per image. The results on all three datasets are given in Table 2. Among the true detection approaches (excluding the MSDS), MSER and HarrLap consistently detect the smallest amount of features (100 to 500) whereas FAST detects a vast amount of features (1,400 to 3,200). Comparing all methods, the grid-based MSDS provides the highest amount of local features, i.e., about an order of magnitude more compared to the true detectors. The multi-scale Hessian and Harris detectors double the amount of local features in comparison to their variants with Laplacian scale detection (HessLap and HarrLap).

**Table 2:** Median amount of local features extracted per image for the OF17, the OF102, and the JF30 dataset.

Detector	local features per image		
	OF17	OF102	JF30
DoG	1,308	972	561
DoH	1,735	1,524	1,473
FAST	3,224	2,519	1,463
MSER	361	343	318
MSDS	12,264	12,348	12,883
Hessian	1,669	1,121	196
HessLap	894	607	110
Harris	1,182	887	316
HarrLap	479	363	109

#### Relevance of affine covariance

In order to evaluate the relevancy of affine covariance for classification, we computed the classification accuracy using the Hessian, HessLap, Harris, and HarrLap with and without affine shape estimation. SIFT was used for descriptor extraction. The FV with 64 codewords was used for encoding and codebooks were computed per detector and split. Hessian and Harris were used on multiple scales whereas the trace of Laplacian was used for scale detection for the Laplacian variants (HessLap and HarrLap). The fixed scale detectors consistently showed poor results compared to their multi-scale or Laplacian variants and were thus excluded from further evaluation. Results show that affine covariance does not substantially impact flower classification accuracies (see Table 3). Instead, slightly worse accuracies were obtained especially on the Oxford Flower datasets. Whereas affine covariant detectors showed high stability against viewpoint changes allowing for good performance in feature matching tasks [37], the affine shape estimation tends to lower the discriminative power of local features upon encoding. Computing time can thus be saved (see Table 2) by using the Harris or Hessian detectors without the need for an iterative affine shape estimation. The Harris corner detector was found to deliver slightly better local features compared to the Hessian detector. Furthermore, the Laplacian variants perform worse compared to the multi-scale variants of the Hessian and Harris detectors, likely caused by the smaller amount of local features.

**Table 3:** Classification accuracy on the OF17, the OF102, and the JF30 dataset. Computed using SIFT in combination with the Hessian and Harris-based detectors without and with (values in brackets) affine shape estimation.

Detector	Classification Accuracy [%]		
	OF17	OF102	JF30
Hessian (HessianAff)	**78.6** (78.0)	**48.4** (46.7)	64.6 (**65.7**)
HessLap (HessLapAff)	**77.1** (77.0)	**44.7** (43.9)	60.7 (**61.2**)
Harris (HarrisAff)	79.2 (**79.9**)	**49.4** (48.8)	**69.0** (67.7)
HarrLap (HarrLapAff)	**75.8** (73.7)	40.4 (**41.0**)	**60.7** (60.3)

**Finding 1**

For image classification, affine covariance of image patches does not significantly impact classification accuracy.

### Extraction

Based on the findings of the previous section, we further evaluated the DoG, DoH, FAST, Hessian, Harris, and MSDS as feature detectors and used them in combination with the local shape descriptors (SIFT, SURF, and HOG) and the color descriptors (RHH, OppA, and DCD). After detecting local features, the local descriptors were extracted and then encoded into a global image representation using the FV encoding. The SIFT, SURF, and HOG codebooks contained 64, 128, and 228 codewords respectively. Codebook sizes were chosen to yield image representations of about 2^14^ elements, a typical size used for shape descriptors (compare with Table 1). The RHH and OppA codebooks contained 15 codewords yielding a 540 dimensional image representation each. The DCD codebook contained 24 codewords yielding a 528 dimensional image representation, as typically used for pure color descriptors (cp. Table 1). Resulting classification accuracies are given in Table 4.

**Table 4:** Class averaged classification accuracy of the studied shape and color descriptors for the OF17, the OF102, and the JF30 datasets.

Classification Accuracy on OF17 [%]						
Detector	Shape Descriptor			Color Descriptor		
	SIFT	SURF	HOG	RHH	OppA	DCD
DoG	75.3	68.1	64.1	54.3	44.7	65.9
DoH	78.6	**81.2**	68.7	52.7	44.9	67.1
FAST	74.4	76.1	69.5	55.4	46.7	68.0
Hessian	78.6	72.6	65.1	52.7	44.9	67.1
Harris	79.2	67.2	62.7	47.7	39.1	59.1
MSDS	68.8	77.9	68.1	50.3	46.8	**69.12**
MSER	63.7	52.7	52.9	43.3	34.0	59.9
Classification Accuracy on OF102 [%]						
DoG	49.0	37.6	36.8	34.5	22.7	40.6
DoH	**59.9**	56.4	39.2	34.0	25.3	42.1
FAST	51.1	58.5	42.3	35.5	25.8	41.9
Hessian	48.4	41.6	32.1	32.0	21.6	39.8
Harris	49.4	37.8	32.1	32.0	20.8	38.5
MSDS	47.2	52.3	44.8	33.6	28.1	**43.7**
MSER	36.8	28.3	25.1	26.3	17.0	32.7
Classification Accuracy on JF30 [%]						
DoG	74.9	72.5	68.5	58.7	66.3	66.3
DoH	**91.6**	88.6	75.7	63.9	71.1	69.5
FAST	79.9	77.6	76.5	60.6	66.2	66.2
Hessian	64.6	65.7	55.9	49.4	52.7	57.3
Harris	69.0	64.0	55.7	50.1	55.6	56.0
MSDS	67.7	79.9	75.1	63.4	**79.7**	71.8
MSER	73.8	64.6	59.7	52.7	54.2	61.9

Whereas MSDS yields high classification accuracy for scene recognition tasks [34], it shows no improvement for our flower classification. Instead, using true local feature detectors such as DoH even improves the classification accuracy achieved while lowering the number of features and hence the computational effort by an order of magnitude. Comparing our results to previous work studying dense-SIFT and MSDS-SIFT, we closely reproduced previous results even with non-segmented images used for our experiments (cp. Table 1). Using dense- or MSDS-SIFT allowed for classification accuracies of about 70% on OF17 whereas distinct color descriptors allowed for about 66%. Furthermore, replacing SIFT by SURF on the OF17 dataset, a classification accuracy of almost 78% was achieved outperforming the MSDS-SIFT by 9% in total. Whereas our results using HOG are close to the results of Nilsback et al. [5, 15] we were not able to reproduce the results achieved by Angelova et al. [18]. Similarly, by testing larger codebooks (up to 8k) we found no improvement in classification accuracy. These results are again in line with the ones of Nilsback [15], but contrary to the results of Angelova et al. [18]. Except for HOG, all local shape descriptors give worse results if computed on a regular grid. Due to the variations in viewpoint and scale in the Oxford Flower datasets, the interest point based methods are clearly more robust [43].

**Finding 2**

For object classification, MSDS provides dispensable amount of local features and can be replaced by true local feature detectors.

Averaged across all detectors and datasets, SIFT yields about 11% higher classification accuracies compared to SURF and 16% compared to HOG, thus resembling the results of [32, 34, 38–40]. We also confirm the finding of Hietanen et al. [34], i.e., SIFT performs worse in combination with its original DoG detector. The combination of the DoH detector and the SIFT descriptor allows for the highest classification accuracies, achieving 59.9% and 91.6% on the OF102 and the JF30 datasets, respectively.

**Finding 3**

Comparing SIFT, SURF, and HOG, the SIFT allows for the highest classification accuracies though it yields lower performance with its original DoG feature detector. The highest accuracy is achieved using the DoH detector.

The Hessian and Harris detectors show results comparable to the FAST and DoG detectors on the Oxford Flowers datasets but worse results on the JF30 dataset, where the average amount of local features is about three times less compared to the OF17 and OF102 dataset (cp. Table 2). Except for the Hessian and Harris detectors, the constraints of the JF30 dataset in terms of image viewpoint and scale improve the classification accuracy, especially for the MSDS approach that is known to be highly sensitive to viewpoint and scale changes. MSER was previously characterized to detect image regions complementary to corners and blobs [41]. In addition to the results summarized in Table 4, we picked the detector allowing for the highest classification accuracies, i.e., DoH, supplemented it with MSER and used SIFT as descriptor. However, none of the classification accuracies improved in this experiment.

Comparing the color descriptors, the DCD descriptors [53] are outperforming the explicitly photometric invariant color descriptors for all detectors by 40% (RHH) and 20% (OppA), respectively. However, given a relative improvement of 11% using MSDS, DCD is slightly outperformed by the OppA color descriptors on the JF30 dataset, which contains large variations in object colors due to the very different illumination conditions during image acquisition. OppA is specifically designed to be invariant against changes in the illuminantion in presence of diffuse light (as in the JF30 dataset), but is still sensitive to changes in the lighting geometry (as in the OF17 and OF102 datasets) [55]. Furthermore, for the viewpoint and scale restricted JF30 dataset, all color descriptors perform 19% better if extracted from grid-points (i.e., MSDS) rather than extracted from detected local features.

**Finding 4**

The DCD allows for high classification accuracy while being implicitly photometric invariant.

### Encoding

**Fig 5:**
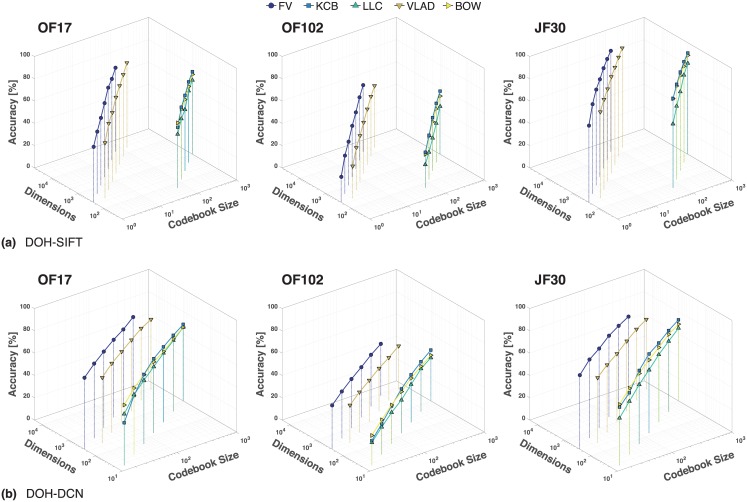
Classification accuracies using (a) DoH-SIFT and (b) DoH-DCD features and different encoding methods for discrete codebook sizes and image representation lengths.

In 2011, Chatfield et al. compared feature encoding methods on the challenging PASCAL VOC 2007 and Caltech-101 datasets [63]. They controlled all parameters of the classification pipeline apart from feature encoding. We followed this approach and extended their work by incorporating the promising VLAD method [60]. Additionally, we were interested in the memory footprint of the resulting descriptors and thus compared the encoding methods in terms of information density by keeping the dimensions of the image representation set to discrete values. Based on the previously discussed results, we selected the best performing detector-descriptor combinations, i.e., DoH for the detection of local features, SIFT for the extraction of local shape descriptors, and DCD for the extraction of color descriptors. The number of codewords was computed to yield discrete image representation lengths of 2^*n*^ with *n* = 8…14 for SIFT descriptors and *n* = 4…10 for DCD descriptors. For BOW, KCB, and LLC we restricted the codebook size to a maximum of 1,024 SIFT codewords. For example, *n* = 8 and 128-dimensional local SIFT descriptors resulted in codebooks containing 256 codewords for BOW, KCB, and LLC, whereas only one and two codewords were computed for FV and VLAD, respectively. Results of the evaluated encoding methods in terms of classification accuracy are shown in Fig 5.

First, our results confirm Chatfield’s et al. finding that larger codebooks lead to higher recognition rates [63]. The performance is not saturated even for high-dimensional image representations and is likely to further improve for larger codebooks. The relative gain using soft-assignment (KCB) against the hard-assignment (BOW) is 2% on average. The overall classification accuracy using LLC is lower compared to BOW. Chatfield et al. concluded that LLC benefits considerably from large codebooks [63].

By comparing encoding methods with each other based on the number of codewords (see Fig 5(a)), FV is the best performing approach, i.e., either 16 (OF17 and JF30) or 32 (OF102) SIFT codewords are sufficient using FV to achieve classification accuracies comparable to KCB with 1,024 codewords. The observed higher classification accuracy is connected to a larger memory footprint since FV and VLAD encode additional statistics within codewords (residuals and covariances). The resulting dimensions of the image representations are multiplied by the dimension of the utilized local descriptor. A comparison based on image representation lengths shows that VLAD and FV require an order of magnitude more elements in the image representation for demonstrating a noticeable effect over other encoding methods. On the other hand, due to the high memory consumption they require increased computational effort for calculating codebooks of the sizes reached by VLAD and FV encodings (more than 1,024 codewords). VLAD and FV allow for high-dimensional and discriminative image representations without demanding huge codebooks. Comparing FV and VLAD for the same image representation lengths, FV outperforms VLAD by 5.5% on average showing that the encoding of second order information (aka codeword covariances) indeed benefits classification performance.

**Finding 5**

Encoding first and second order statistics (VLAD and FV) increases classification accuracy without demanding large codebooks, thus lowering the computational effort for codebook generation.

### Fusion

For the evaluation of the three fusion strategies, we applied the DoH method for local feature detection as we found it to provide highest classification accuracies (cp. section *Extraction*). Based on the detected local features, we compared the following descriptor-fusion combinations: the earliest fused OpponentSIFT to early and late fusion of the SIFT shape descriptor with the DCD color descriptor. FV was used for encoding local features as it allowed for the best classification accuracies in the previous experiment.

For earliest fusion, SIFT descriptors were computed for every channel of the color image in opponent colorspace. The local descriptors thus contained 384 elements and were encoded using FV with only 22 codewords to yield image representations of dimensions comparable to those in section *Extraction*. Please note that Chai et al. used an order of magnitude more codewords solely for FV encoding of SIFT descriptors on segmented images [19]. For early fusion, we concatenated the local SIFT descriptors with the 11-element DCD descriptors, thus yielding local descriptors of 139 elements. The weight parameter *λ* for the shape descriptor was set to 0.375. These local descriptors were encoded by FV using 59 codewords, again for yielding image representation sizes comparable to the other experiments. Following the late fusion strategy, we concatenated a DoH-SIFT based global representation encoded by FV with 62 codewords and a global MSDS-DCD based representation, also encoded by FV with 24 codewords. The results of the three different strategies are displayed in Table 5.

**Table 5:** Class averaged classification accuracies for the fused shape and color descriptors on the OF17, the OF102, and the JF30 datasets all using the DoH detector.

Fusion strategy	Methods	Classification accuracy [%]		
		OF17	OF102	JF30
earliest	DoH-OpponentSIFT	**91.8**	**72.8**	**94.8**
early	DoH-SIFT+DCD	90.3	70.2	93.2
late	DoH-SIFT+MSDS-DCD	88.1	66.0	91.6

Bianco et al. compared earliest and late fusion for pairwise feature matching on several datasets. Their findings imply that the performance of different fusion techniques depends on the dataset [43]. We found that the earliest fusion (OpponentSIFT) outperforms the other fusion methods by 4% on average, followed by the early fusion strategy. The loss of spatial correspondence between local color and shape information by late fusion results in a relative drop of classification accuracy by 3% compared to early fusion.

**Finding 6**

Loosing spatial correspondence between local color and shape information by late fusion negatively impacts the classification accuracy. Earliest fusion by channel-wise descriptor concatenation allows for the highest classification accuracy.

### Pooling

In order to investigate the impact of spatial binning along with feature pooling, we compared classification accuracies achieved using DoH-OpponentSIFT features on a single-layered image representation to the spatial pyramid representation introduced by Lazebnik et al. [31]. Instead of region-size dependent normalization, we used L2 normalization of the subimage representations. Max-pooling was used for LLC and sum-pooling for BOW and FV encoding [63]. Furthermore, the same codebooks as in the previous section were used, i.e., 1,024 codewords for BOW and LLC as well as 22 codewords for FV. We investigated an increasing amount of pyramidal levels, i.e., one level (1x1), two levels (1x1, 2x2) and three levels (1x1, 2x2, and 4x4). Due to concatenation of the subimage representations, the size of the final image representation is multiplied by the number of subimages. Hence, for BOW and LLC encoding and 1,024 codewords on three pyramidal levels, the final image representation contains about 21.5k elements whereas about 355k for FV, causing larger memory demands and longer training times.

A general finding is that the standard one-level FV encoding consistently outperforms all other encodings (cp. Table 6). This finding is consistent with the results of the encoding experiment in section *Encoding*. The classification accuracy for FV was consistently reduced by using a pyramidal image representation. Using two levels instead of one allowed for slight improvements in case of BOW and LLC on the OF102 and the OF17 dataset. However, by using a three-level pyramid the resulting classification accuracies were the lowest across all datasets. Hence, with respect to the discriminative power and length of the final image representation we found the encoding of first and second order feature-codeword statistics using the FV to be more beneficial for flower classification than adding weak geometric context by using a spatial pyramid.

**Table 6:** Class averaged classification accuracies on the OF17, the OF102, and the JF30 datasets using DoH-OpponentSIFT and an increasing amount of pyramidal levels (one to three).

Pyramidal bins	Encoding	Classification accuracy [%]		
		OF17	OF102	JF30
1x1	BOW	86.0	63.9	94.6
	LLC	85.0	63.2	94.5
	FV	**91.8**	**72.8**	**94.8**
1x1+2x2	BOW	86.0	64.1	94.1
	LLC	86.0	65.1	94.5
	FV	88.8	70.4	93.8
1x1+2x2+4x4	BOW	83.7	63.0	93.3
	LLC	85.6	64.1	93.7
	FV	87.5	68.4	93.2

**Finding 7**

For flower classification, encoding first and second order feature-codeword statistics using FV is more beneficial than adding weak geometrical context by using a pyramidal image representation.

## Conclusion

We performed a comprehensive comparison of state-of-the-art methods within an image classification pipeline for flower image based plant species classification using local features. Hence, we investigated methods relevant for local feature detection, descriptor extraction, encoding, pooling, and fusion. We investigated the impact of the selected methods measured in terms of classification accuracy on three different datasets: the Oxford Flower 17, the Oxford Flower 102, as well as our own Jena Flower 30.

Whereas dense sampling of local features in combination with segmentation or weighted feature pooling is widely used, our results show that comparable or better classification accuracy can be achieved by using true feature detectors. Although enabling high accuracy in scene recognition tasks, multi-scale dense sampling achieved only poor results on our datasets, especially for those with variation in viewpoint and scale, i.e., the Oxford Flower datasets. Additionally, true feature detectors produce at least an order of magnitude less features compared to dense sampling, thus lowering computational effort during descriptor extraction and encoding. The Hessian-based SURF (DoH) detector in combination with SIFT as local shape descriptor was found to be superior over other detector-descriptor combinations in terms of classification accuracy. SIFT was found to perform relatively bad in combination with its original detector (DoG). Comparing the Harris corner and Hessian blob detectors with and without affine covariant image patches, no significant impact on the classification accuracy, rather we gained slightly worse accuracies if affine covariant patches are used. Furthermore, despite the MSER region detector was previously found to detect local features most complementary to corner and blob detectors, the classification accuracy was not found to be improved if it was used as a supplementary feature detector.

With respect to local shape descriptors, we found SIFT to facilitate more accurate classifications compared to SURF and HOG. On the other hand, whereas the overall accuracy using dense sampling was poor, dense sampled SURF features instead of SIFT improved the classification accuracies for all datasets.

Color bagging using a generic but highly discriminative codebook, i.e., the Discriminative Color Descriptor (DCD), was found to be the best local color descriptor in terms of classification accuracy and photometric stability. However, if the dataset covers very different illumination conditions, the performance of the Discriminative Color Descriptor is reduced and the explicitly photometric invariant Opponent Angle descriptor achieves slightly better results, though it generally performs worse.

Concerning feature encoding, we compared the traditional Bag of Words (BOW) to its soft-encoding variants as well as to first and second order encoding methods (VLAD and Fisher Vector). Whereas the Locality Constrained Linear Coding was found to require significantly larger codebooks (> 1k codewords), the Kernel Codebook encoding slightly outperformed the BOW for all codebook sizes. As the generation of huge codebooks requires significant computational resources, the VLAD and FV encoding showed their advantageous utilization of additional visual word statistics that allow to lower the required codebook sizes by at least an order of magnitude for achieving highly discriminative image representations. VLAD and FV were found to perform almost equally well, with FV performing slightly better for larger codebooks.

Comparing different pipelines for fusion of color and shape descriptors before classification revealed that earliest fusion, i.e., OpponentSIFT, achieved the best classification accuracies compared to the early fusion (local concatenation of SIFT and DCD). Late fusion after encoding was found to decrease accuracy due to the loss of spatial correspondence between color and shape information, especially if large variations in viewpoint and scale are present within the dataset.

For scene classification, using a pyramidal image representation increases the classification accuracy by adding weak geometric context, hence it is a widely accepted method for object classification as well. However, we showed that it is more efficient for flower classification to pool and encode local features globally instead of using a pyramidal image representation, especially if the FV is used for encoding.

Whereas the Oxford Flower 17 and especially the Oxford Flower 102 dataset are somewhat challenging as the images represent fairly uncontrolled image situations, the results on our own Jena Flower 30 dataset confirm the benefit of defining constraints in image viewpoint and scale. Being incorporated in applications for mobile devices, such requirements can be easily met and help non-expert users in classifying unknown plant species. Finally, we provide the Jena Flower 30 dataset consisting of images acquired throughout a whole season, thus capturing flowers in visually very different blooming stages and under very different illumination conditions. Despite the higher amount of classes (plant species) compared to the OF17 dataset, high classification accuracies of 94% were realized already for this dataset with a well-designed classification pipeline based on local features.
