# Correction: Plant species classification using flower images—A comparative study of local feature representations

**DOI:** 10.1371/journal.pone.0175101

**Published:** 2017-03-29

**Authors:** The PLOS ONE Staff

There are errors in the Author Contributions. The correct contributions are: Conceptualization: MS PM. Data curation: NA MS. Funding acquisition: PM JW. Investigation: MS MR. Methodology: MS PM. Project administration: PM JW. Resources: PM. Software: MS NA. Supervision: PM JW. Visualization: MS. Writing–original draft: MS. Writing–review & editing: MS PM MR JW.

The following information is missing from the Funding section: We acknowledge support for the Article Processing Charge by the German Research Foundation and the Open Access Publication Fund of the Technische Universität Ilmenau.

The publisher apologizes for the errors.
